# Forecasting Seizures Using Intracranial EEG Measures and SVM in Naturally Occurring Canine Epilepsy

**DOI:** 10.1371/journal.pone.0133900

**Published:** 2015-08-04

**Authors:** Benjamin H. Brinkmann, Edward E. Patterson, Charles Vite, Vincent M. Vasoli, Daniel Crepeau, Matt Stead, J. Jeffry Howbert, Vladimir Cherkassky, Joost B. Wagenaar, Brian Litt, Gregory A. Worrell

**Affiliations:** 1 Mayo Systems Electrophysiology Laboratory, Mayo Clinic, Rochester, MN, United States of America; 2 Veterinary Medical Center, University of Minnesota, St. Paul, MN, United States of America; 3 School of Veterinary Medicine, University of Pennsylvania, Philadelphia, PA, United States of America; 4 NeuroVista Inc. Seattle, WA, United States of America; 5 Department of Electrical and Computer Engineering, University of Minnesota, St. Paul, MN, United States of America; 6 Penn Center for Neuroengineering and Therapeutics, University of Pennsylvania, Philadelphia, PA, United States of America; Radboud University Nijmegen, NETHERLANDS

## Abstract

Management of drug resistant focal epilepsy would be greatly assisted by a reliable warning system capable of alerting patients prior to seizures to allow the patient to adjust activities or medication. Such a system requires successful identification of a preictal, or seizure-prone state. Identification of preictal states in continuous long- duration intracranial electroencephalographic (iEEG) recordings of dogs with naturally occurring epilepsy was investigated using a support vector machine (SVM) algorithm. The dogs studied were implanted with a 16-channel ambulatory iEEG recording device with average channel reference for a mean (st. dev.) of 380.4 (+87.5) days producing 220.2 (+104.1) days of intracranial EEG recorded at 400 Hz for analysis. The iEEG records had 51.6 (+52.8) seizures identified, of which 35.8 (+30.4) seizures were preceded by more than 4 hours of seizure-free data. Recorded iEEG data were stratified into 11 contiguous, non-overlapping frequency bands and binned into one-minute synchrony features for analysis. Performance of the SVM classifier was assessed using a 5-fold cross validation approach, where preictal training data were taken from 90 minute windows with a 5 minute pre-seizure offset. Analysis of the optimal preictal training time was performed by repeating the cross validation over a range of preictal windows and comparing results. We show that the optimization of feature selection varies for each subject, i.e. algorithms are subject specific, but achieve prediction performance significantly better than a time-matched Poisson random predictor (p<0.05) in 5/5 dogs analyzed.

## Introduction

Epilepsy afflicts over 50 million people worldwide, and is second in prevalence only to stroke among debilitating neurological conditions [1, 2]. Many people with epilepsy (PWE) do not achieve complete seizure control with medication, and even following resective epilepsy surgery seizures may persist. The constant threat of an unexpected, seizure often prevents PWE from participating in many daily activities [3]. This, in addition to the potential psychological impact makes it challenging for PWE without complete seizure control to live fully satisfying lives.

An accurate seizure warning system (SWS) could allow patients to modify activities to avoid risk, or take additional medications to prevent seizures. In order to predict seizures, robust methods for identifying iEEG patterns that precede a patient's habitual seizures are needed. There is emerging evidence for a consistent sequence of local field potential (LFP) patterns preceding and leading into seizures in some patients[4], and it has been hypothesized that seizures may arise from identifiable brain states. Numerous clinical studies describe patients self-reporting seizure prone states hours or days prior to seizure [5] at a rate greater than random chance [6]. Changes in cerebral blood flow, oxygenation, and cortical excitability have also been measured preceding seizures [7–11].

While many early seizure prediction studies suffered from inadequate statistical rigor, [6, 12], more recent reports have been successful using more rigorous statistical approaches [13–18]. A persistent difficulty in assessing seizure prediction algorithms is the scarcity of long duration recordings with an adequate number of spontaneous seizures and duration of interictal data. Presurgical human iEEG recordings typically last less than 10 days due to the discomfort and risk associated with invasive iEEG recordings [19, 20]. These recordings exhibit iEEG changes due to rapid tapering of antiepileptic drugs (AED) [21, 22], and they rarely contain an adequate number of seizures separated by sufficient time to permit adequate statistical characterization of both the preictal and interictal periods. Longer-duration iEEG recordings are possible in animals, though typically from models of epilepsy where an epileptic focus has been artificially created by introduction of a systemic or topical pharmacological agent, [23] or traumatic injury [24]. The applicability of these induced animal models to forecasting habitual seizures in naturally occurring human epilepsy is unclear.

Naturally occurring canine epilepsy is an excellent model for human epilepsy [25, 26]. Canine epilepsy occurs at the same rate and is resistant to drug therapy at the same rate as human epilepsy [26]. The clinical [27] and electrophysiological [28, 29] characteristics of canine epilepsy are very similar to focal human epilepsy, and canine and human iEEG of focal onset seizures essentially indistinguishable [30]. Many medications used to treat human epilepsy, e.g. phenobarbitol and leviteracitam, are also effective in canines at similar serum levels [31–34]. Canines are large enough to test devices designed for humans [30, 35], are capable of safely tolerating electrode implantation [36], and are a widely available and inexpensive animal model for research [26]. Canine epilepsy represents a close analog to human epilepsy, and is capable of providing prolonged ambulatory iEEG recordings under tightly controlled conditions not possible with human subjects.

Recently a clinical pilot study of seizure forecasting was performed by researchers in Australia and NeuroVista Inc. using chronic ambulatory recordings in 15 human patients [15] [30, 35]. The patient cohort in this study had between 2 and 12 seizures per month and the device achieved greater than 65% sensitivity in 11/15 patients during the training phase with a mean (standard deviation) 27.8 (±11.6)% of time spent in high-likelihood seizure warning. Performance degraded during the 4-month prospective portion of the study to 4/14 patients with seizure forecasting sensitivity greater than 65% with 23.0 (±11.2)% of the total time in seizure warning. This study successfully demonstrated the safety and practical feasibility of seizure forecasting in some humans with focal epilepsy [37], although continued improvement in forecasting sensitivity and specificity (time in warning) are likely needed to make the system clinically useful.

Subsequently, our group in conjunction with the NeuroVista team reported successful seizure forecasting in three dogs with naturally occurring epilepsy implanted with the same NeuroVista SAS device [18]. This study used a logistic regression machine learning algorithm with spectral power in the traditional Berger bands as features, and achieved lead (> 4 hour separation) seizure prediction rates greater than a time-matched chance predictor [13] in 2/3 dogs. The present study extends and expands these results in a larger cohort of dogs. This manuscript describes the development and validation of a support vector machines (SVM) approach to seizure forecasting using power in band (PIB) and inter-electrode synchrony features calculated from prolonged, ambulatory iEEG recordings from canines with naturally occurring epilepsy [18, 35]. The optimal preictal time window for seizure forecasting was investigated, and analysis of the impact of multiple PIB features and individual electrode pairs on inter-electrode synchrony features was performed. We show that the optimal iEEG feature set varies for each subject, i.e. algorithms are subject specific, but that prediction performance significantly better than a time-matched Poisson random predictor (p<0.05) was possible in all 5 of the dogs analyzed.

## Materials and Methods

**Fig 1:**
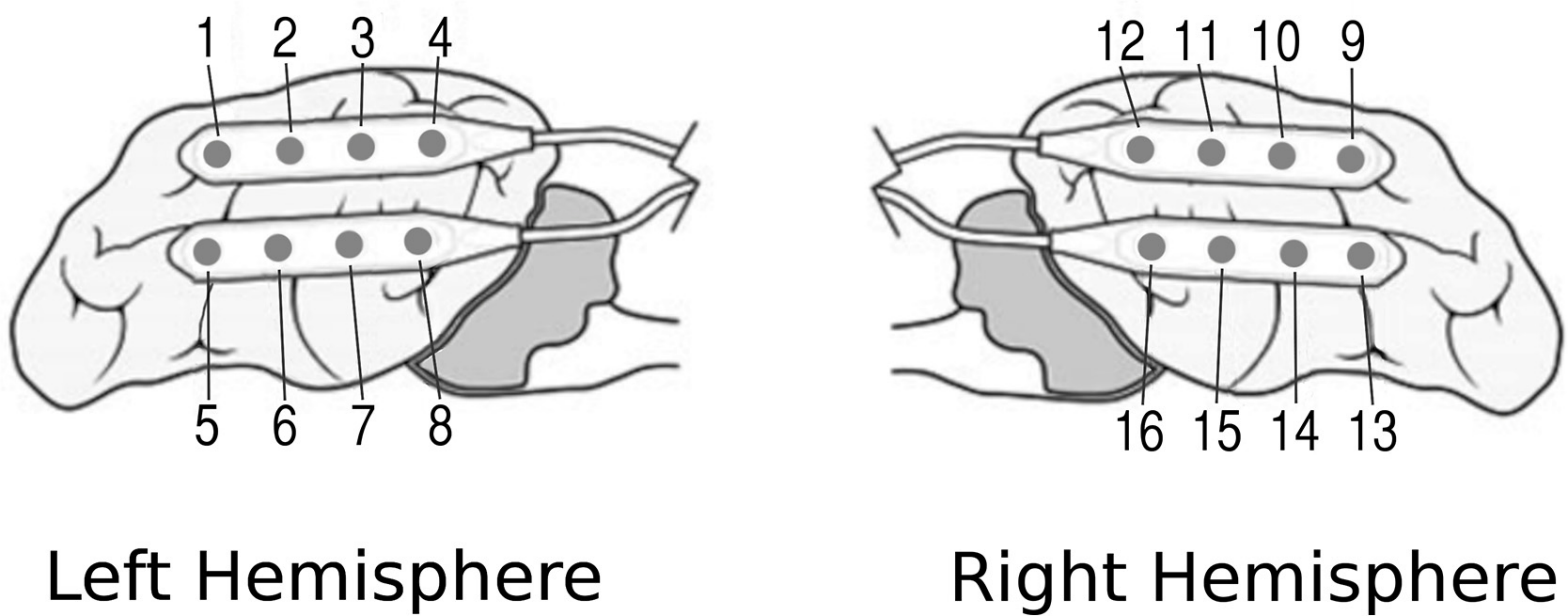
Approximate placement and numbering of sixteen implanted electrode contacts relative to the canine cortical anatomy.

This study involved eight canines with naturally occurring epilepsy implanted with mobile intracranial EEG monitoring devices described previously [30, 35]. The implanted telemetry device records iEEG data at 400Hz from a bilateral array of sixteen electrodes (Fig 1) with an average reference, and transmits data wirelessly to a data storage device in a vest worn by the dog. Demographic and technical details describing the canine recordings are presented in Table 1. Of the eight canines three were excluded from analysis due to an inadequate number of recorded seizures. Canines were housed and cared for at the Veterinary Medical Centers at the University of Minnesota and University of Pennsylvania. None of the dogs were on antiepileptic medication at the start of the study. For device implantation canines were given established doses of acepromazine, morphine, and propafol for anesthesia, with fentanyl provided for additional pain control. Dopamine (to increase blood pressure and increase cerebral blood flow) and lactated Ringer’s solution was given intraoperatively as needed. Cefazolin was administered for infection control before and after surgery. Electrode strips were placed via bilateral craniectomies and anchored caudally with a silicone lead anchor. Lead wires were tunneled through caudal holes in the craniectomies, looped anteriorly and anchored to the frontal bone with a titanium screw, and then tunneled under the skin to the telemetry unit placed under the latissimus dorsi muscle. Polymethyl methacrylate gelfoam was used to seal any cranial openings. Postoperatively radiographs were acquired to confirm proper electrode placement, buprenorphine was administered as needed for pain control, and acepromazine sedation was given as needed to prevent self-injury. After nearly one year of continuous data acquisition one canine was humanely euthanized following observation of progressive ataxia and neurological decline followed by respiratory arrest. Postmortem examination revealed bleeding near a secondary implant surgery that had recently been performed. Dogs in the study were monitored continually [35], and all canine care and treatment protocols used in this study were approved by IACUC review boards at Mayo Clinic, University of Minnesota, and University of Pennsylvania.

**Table 1:** Testing data.

*Dog Number*	*Dog Name*	*Date Implanted*	*Recording Begin Date*	*Recording End Date*	*recording duration (days)*	*recording duration without gaps (days)*	*annotated seizures*	*lead seizures*
1	Buck	7/30/09	7/30/09	11/18/10	476	342	47	40
*2*	*Tanner*	*7/15/09*	*7/15/09*	*11/18/10*	*398*	*255*	*2*	*2*
3	Drools	8/27/09	8/27/09	11/22/10	452	213	104	18
4	Foster	5/7/12	5/8/12	6/5/13	393	298	29	27
*5*	*Gus*	*5/8/12*	*5/9/12*	*4/12/13*	*338*	*29*	*0*	*0*
6	Joseph	5/14/12	5/15/12	2/26/13	287	168	144	86
7	Ripley	5/15/12	5/16/12	3/6/13	294	80	22	8
*8*	*Sakic*	*5/16/12*	*5/22/12*	*3/8/13*	*290*	*126*	*0*	*0*

Eight mixed-breed canines with naturally occurring epilepsy were implanted with a mobile iEEG recording device and monitored continuously for multiple months. Four dogs had an inadequate number of seizures for algorithm training and testing. Lead seizures are defined as seizures separated by a minimum of 4 hours. Dogs with fewer than 5 lead seizures (italicized) were excluded from analysis. Dog 1 (Buck) died after approximately a year of iEEG monitoring.

Recorded iEEG on the data storage device were transferred to a central repository via a cloud-based data storage service. Data were translated into multiscale electrophysiology format (MEF) [19] and bandpass filtered into 11 non-overlapping contiguous frequency bands (0.61–3.8 Hz, 3.8–9.7 Hz, 9.7–18.2 Hz, 18.2–29.5 Hz, 29.5–43.5 Hz, 43.5–60.2 Hz, 60.2–79.5 Hz, 79.5–101.6 Hz, 101.6–126.4 Hz, 126.4–153.9 Hz, 153.9–184.1 Hz) using a finite impulse response Bartlett-Hanning window with 4194304 points. Frequency bands were chosen in order to characterize the frequency response of correlation across the relevant range of recordable frequencies with this device at finer frequency resolution than the conventional EEG frequency bands used in prior studies [14, 18, 46]. Frequency bands between 184.1 Hz and the Nyquist limit appeared to have some sampling artifact contamination and were avoided. Correlations between physically adjacent contacts within each 4-contact strip (Fig 1) were calculated on the filtered signals and summed into one-minute bins. This produced 12 correlations per dog (3 per strip) in each of the 11 frequency bands, creating a classification space of 132 features. We also computed univariate spectral power in frequency band features as described in [18] [18](0.1–4 Hz, 8–12 Hz, 12–30 Hz, 30–70 Hz, 70–180 Hz) and tested these features separately using an SVM classifier for comparison. For all dogs the data recorded within 70 days of electrode implantation was excluded from analysis due to observed large-scale non-stationarity in the iEEG following surgery. Classification of interictal and preictal epochs was performed using PIB and inter-electrode synchrony iEEG features and the open-source libSVM implementation [38] of the support vector machines (SVM) machine learning algorithm. We used the C-SVC SVM type with a linear kernel, and category weights inversely proportional to the ratio of the number of training samples. The kernel function gamma and cost parameters were tuned using a small excerpt of canine training data with a grid search python utility supplied with the libSVM distribution.

An overlapping window approach was used to aggregate individual one-minute bin classifications and trigger seizure warnings. Preictal bin classifications within a moving window equal in length to the preictal duration (typically 90 minutes) are summed, and a seizure warning equal in duration to the preictal window is initiated if the number of preictal bins exceeds a tunable threshold. [39] A five-fold cross validation method was applied, where the available iEEG data was divided into five equal-length portions, four of which were used for training, with one segment held out for assessment. Each data segment was held out in turn for testing, and testing results were assembled to provide an assessment covering the entire recording. We generated receiver operating characteristic (ROC) curves by varying the window threshold. In this application, sensitivity is simply the proportion of lead seizures that occur while the algorithm is in a warning state. Previous authors have formulated specificity rigorously as the proportion of monitored time not spent in a warning state [13], and we follow that convention here. Hence we describe the independent axis (1-specificity) by the term "time in warning" (TIW), representing the proportion of the recording in a warning state. Statistical significance was determined at a p<0.05 level calculating p-values relative to a time-matched Poisson random predictor as described in [13].

The preictal window used for classification has varied among prior studies with little physiological justification given. In the present study a 90-minute preictal period was initially used for comparison with prior long-duration iEEG studies, after which the preictal data interval was increased from 10 to 240 minutes by 10 minute intervals. We hypothesize that the algorithm's performance will be optimized when the preictal classification window most closely matches the length of the true physiological preictal signature, as this maximizes available training data without including interictal data points in the preictal training set. Results for this experiment are reported as seizure prediction sensitivity at 30% TIW to maintain a consistent comparison.

Prior studies have followed the traditional frequency ranges with only minor variation. While this system separates frequency components into clinically familar ranges, there is little empirical or theoretical justification for this system being relevant to seizure forecasting. We hypothesize that the preictal signature is composed of specific frequency components, and that removal of extraneous frequency components will improve classifier performance by reducing overfitting [40]. To test this hypothesis we tracked classification performance for each individual frequency band, while omitting single frequency bands, and while including increasing frequency bands from near 0.6 Hz to the Nyquist limit. Our highly specific choice of frequency bands permits finer sampling especially in the high frequency ranges, as these high frequency features were often significant in prior studies [14]. Results for this experiment are also reported as sensitivity at 30% TIW.

The sixteen-channel implanted electrodes provide good general coverage of the canine brain, but no data exists addressing the number and arrangement of electrodes needed for adequate seizure forecasting. To investigate this we repeated the basic seizure forecasting experiment on inter-electrode pairs from different hemispheres. Given the sparse placement of electrodes we were unable to identify a clear seizure onset zone from the dogs’ iEEG, and therefore no attempt was made to analyze seizure onset electrodes separately or in relation to other electrodes.

## Results

**Fig 2:**
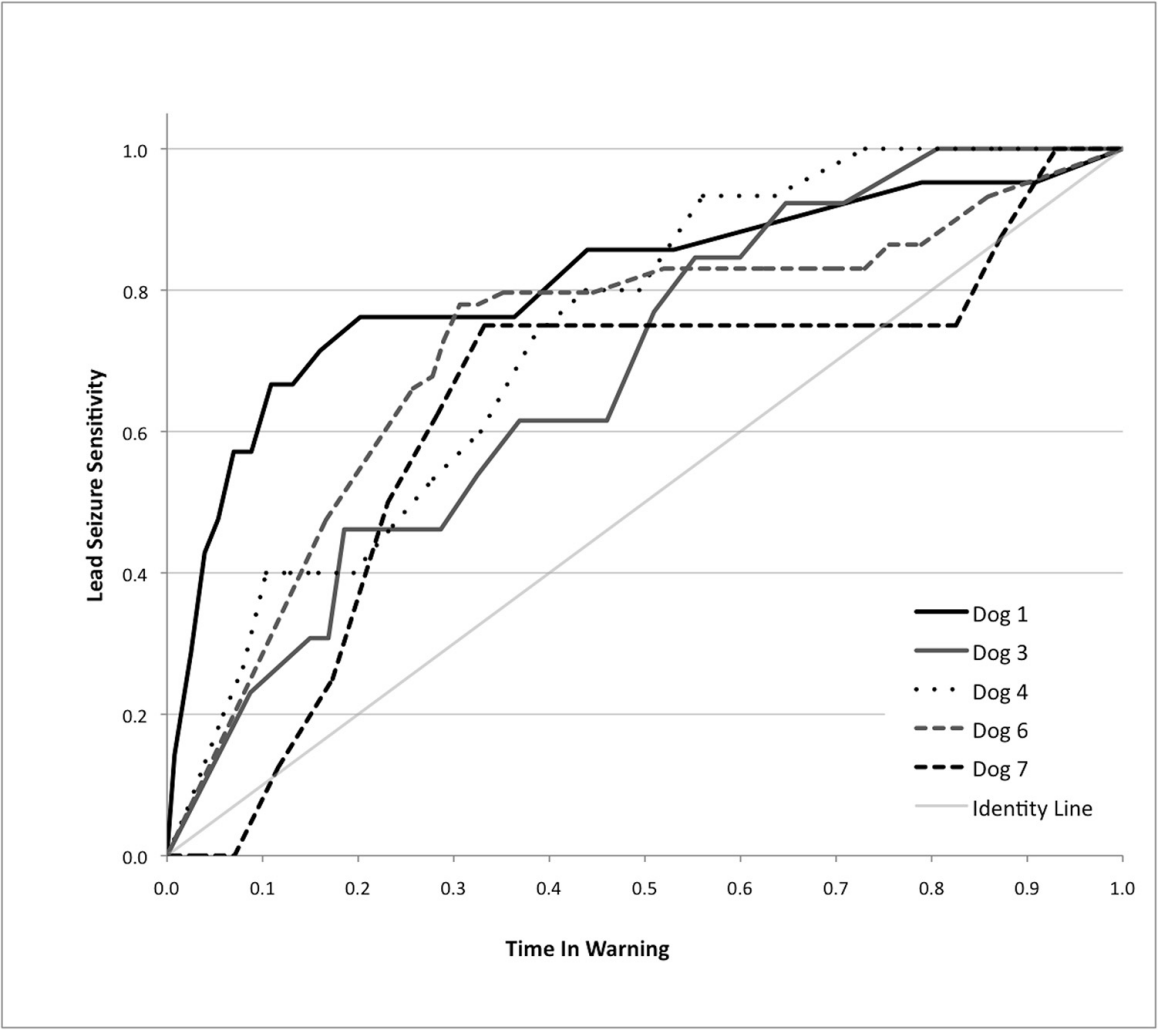
Receiver-operating characteristic curves for the five analyzed canines. Curves were generated by varying the threshold required to initiate a seizure warning.

Receiver operating characteristic (ROC) curves using a 90-minute preictal window with 16 electrodes and all frequency bands were generated by varying the window threshold after SVM classification (Fig 2). Mean area under the curve (AUC) was 0.72. Using the adjacent inter-electrode correlation feature at 30% TIW the results were statistically significant for all dogs except for dog 3. Results using spectral band power were also significant at 30% TIW for the same four dogs, with better lead seizure sensitivity than correlation in 3 dogs and worse sensitivity in 2 dogs. (Table 2) The optimal seizure forecasting performance was obtained with the use of inter-electrode correlations that spanned both hemispheres (Table 3).

**Table 2:** 90 minute preictal window targeting 30% time in warning.

*Dog*		Correlation					Power In Band			
	*TIW*	*FP/D*	*DWW*	*Lead Sn*	*p*	*TIW*	*FP/D*	*DWW*	*Lead Sn*	*p*
1	0.25	0.90	86	0.76	< 0.001	0.29	1.40	79	0.80	< 0.001
3	0.29	0.64	109	0.46	0.115	0.29	0.42	130	0.30	0.496
4	0.28	1.04	74	0.53	0.025	0.30	1.16	97	0.81	< 0.001
6	0.29	0.19	63	0.73	< 0.001	0.30	0.19	60	0.66	< 0.001
7	0.28	0.84	38	0.63	0.038	0.30	0.96	37	1.0	0.019

Results of SVM classification of correlation (left) and spectral power in band (right) features for the five canines with adequate data and number of seizures to permit training and testing. To facilitate comparison the algorithm was tuned to approach 30% time in warning. TIW (time in warning) represents the proportion of the recording the algorithm labeled as preictal. FP/D (false positives per day) describes the mean number preictal warnings that did not produce seizures. DWW (days without warning) represents the number of 24-hour periods in which no preictal warning occurred. Lead Sn (sensitivity) represents the proportion of lead (>4 hour separation) seizures successfully predicted by the algorithm. The p-value was calculated using the formulation in [13].

**Table 3:** Bilateral electrode pairs improve performance.

*Dog*	*Location*	*TIW*	*FP/D*	*Days w/o warn*	*Sensitivity*	*p*
1	Ant-inf	0.300	1.735	55	0.857	0.000
3	Post-inf	0.300	0.606	122	0.571	0.022
4	Center-sup	0.297	1.427	53	0.696	0.000
6	Center-sup	0.299	0.293	61	0.706	0.000
7	Post-inf	0.283	1.452	20	0.625	0.038

Lead seizure sensitivity at 30% TIW with correlation features improves if the classifier is restricted to specific bilateral electrode pairs, suggesting the iEEG preictal signature is not homogeneously distributed across the brain.

**Fig 3:**
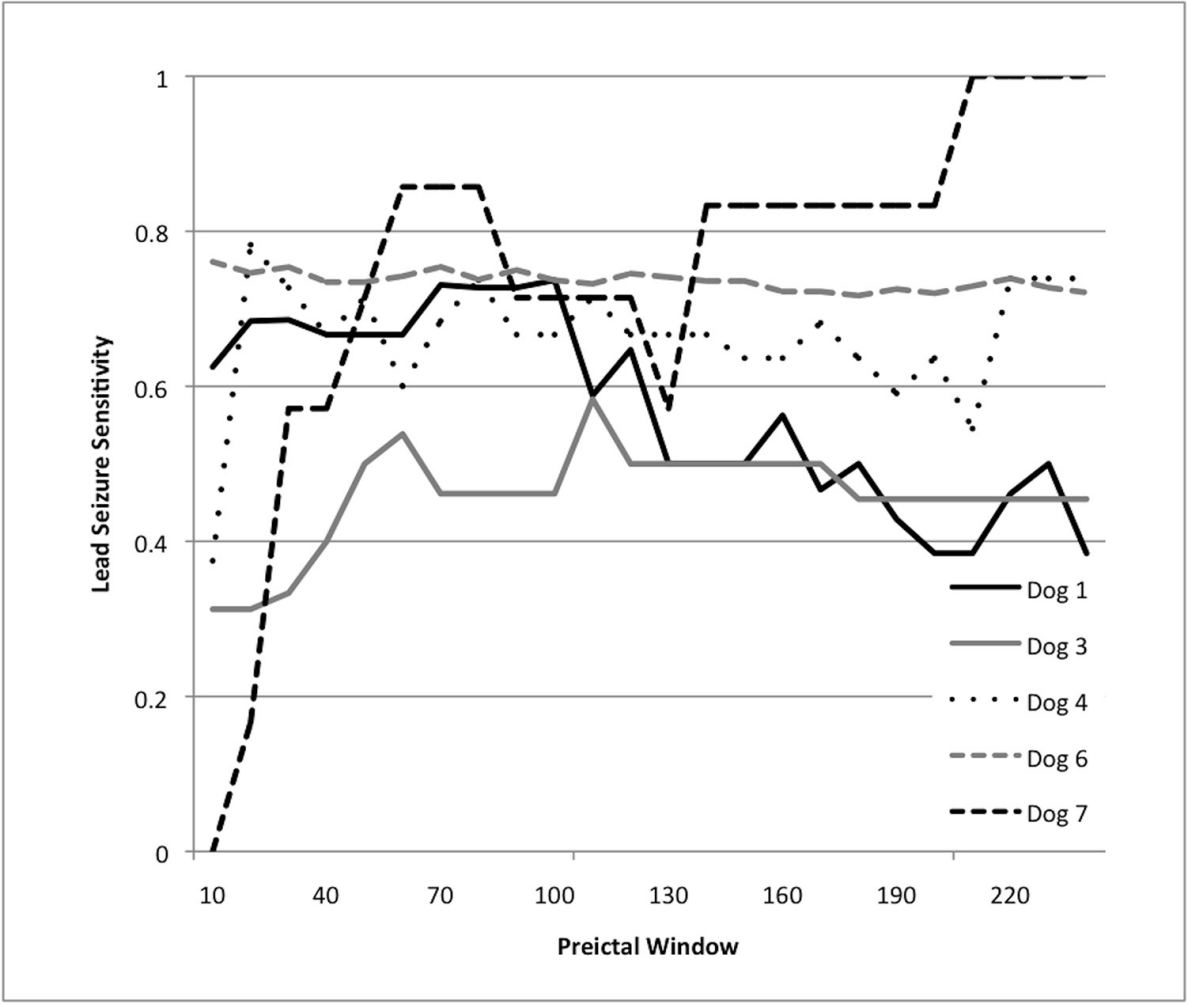
Performance of the SVM-correlation seizure prediction method varies with the choice of the preictal training window. The horizontal axis scales the preictal analysis window in minutes, and the vertical axis shows lead seizure sensitivity for the algorithm, if the algorithm threshold is tuned to maintain time in warning at 30%.

The dependence of preictal window size at which the classifier achieved peak performance (Fig 3) varied between different subjects, and many of the dogs (e.g. 3,7) exhibited bimodal or multimodal performance. Interestingly, Dog 6 showed very little variation with preictal window size, while Dog 7’s variation spanned the entire range of sensitivity.

**Fig 4:**
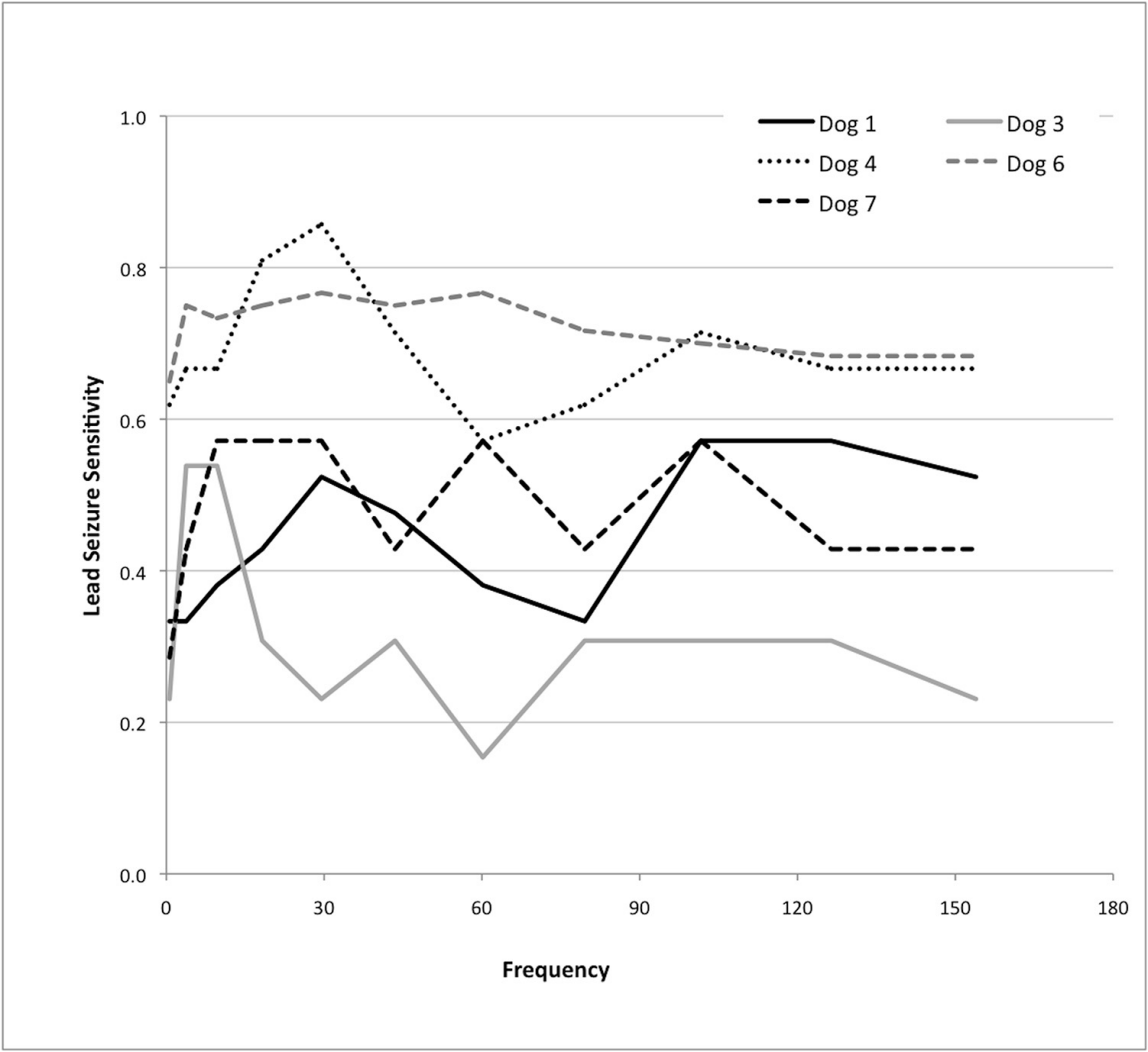
Performance of the SVM-correlation seizure prediction method varies with changes in the frequency band analyzed. The horizontal axis shows the frequency band analyzed in hertz, while the vertical axis shows lead seizure sensitivity for the algorithm, if the algorithm threshold is tuned to maintain time in warning at 30%.

The results of analysis of single frequency bands are shown in Fig 4. The best performing correlation frequency range was dog-specific, but frequencies below 43.5 Hz performed well for all dogs.

For dogs 1, 3, and 4 at least one set of bilateral correlation pairs resulted in better forecasting performance than the entire set of electrodes (Table 3). For dog 6 forecasting performance declined slightly, while for dog 7 performance was unchanged. Forecasting for all dogs with the best bilateral electrode set was significantly better than a time-matched chance predictor [13].

## Discussion

This study expands upon work previously reported by our group [18] using univariate power-in-band features with a logistic regression classifier in three canines. Using a support vector machines classifier provides a more powerful machine learning approach for incorporating multiple features [41] for the seizure forecasting problem [42–44]. Bivariate features have shown promising results in prior seizure forecasting studies [45] and have been shown to be a biomarker of seizure onset zone [46]. The results of the present study support the idea that inter-electrode correlation may be an indicator of seizure generation in focal epilepsy. While it seems possible to improve performance by analyzing inter-electrode synchrony in relation to the seizure onset zone, we were unable to delineate a clear and consistent seizure onset zone in any of the dogs studied which is likely due to the generic placement of electrodes. In future studies it may prove useful to perform imaging and iEEG studies to identify seizure onset zone prior to prolonged, ambulatory iEEG monitoring. The variation in bilateral pair performance and single frequency band correlation performance highlights the variation between subjects in preictal iEEG characteristics and, from a data-analytic perspective, the need for subject specific predictive models. Comparison with the spectral power features suggests that correlation and spectral power may both be valuable for seizure forecasting, and the relative performance of the two feature sets also appears to be subject dependent. These results suggest subject-specific tuning of the prediction model to specific features may be useful for reducing the dimensionality of the classification space.

In order to be reliable enough for patients to plan and schedule daily activities, seizure forecasting must attain high sensitivity while maintaining a reasonably low rate of false positives. In this study we tuned our algorithm to maintain approximately 30% TIW to facilitate comparison between experiments. TIW is an imperfect metric of specificity, as it counts warnings that preceded seizures against specificity, and doesn’t entirely represent the potential impact of false positives on a patient’s lifestyle. For example, multiple single false warnings distributed over a few days are likely to be more disruptive than a single continuous false warning that persists for multiple hours on a single day. Tables 2 and 3 report results including “Days Without Warning” (DWW), which is the number of 24-hour periods in which no seizure warning occurred. This represents the number of days during the study in which a patient may not have needed to take medications, reducing overall AED dose and attendant side effects [47, 48].

This manuscript describes seizure forecasting results better than a time-matched Poisson random predictor [13] in long duration iEEG recordings from dogs with naturally occurring epilepsy. While the iEEG datasets described in this manuscript are unparalleled in length and quality, the limited data sampling rate (400 Hz) restricts the analysis to conventional frequency bands and precludes studying the potential value of ripple and fast ripple oscillations [49, 50] in seizure forecasting. A fundamental limitation of the current study is the benchmark against a Poisson predictor. Seizures often cluster and there may be temporal dependencies that extend beyond our requirement that all analyzed seizures were at least 4 hours apart. In addition, the known diurnal variation of the iEEG and association of seizures with the sleep wake cycle are not addressed in the current study. An additional challenge in the analysis of this data is the potential diurnal inductive noise in the data resulting from the need to charge the recording unit's battery daily. Because the exact timing and duration of battery charging was not available, we were unable to correct or filter this effect. Further, due to inevitable occasional equipment maintenance and failures, some data loss, showing up as gaps in the recording, occurred in these recordings. While most of these data gaps are small (on the order of a few minutes or a few hours) in a few cases gaps of multiple weeks occur in the data. In our analysis any recording gaps greater than one hour were treated as potential seizure events, and the one week exclusion for interictal data was applied.

A major challenge in seizure forecasting studies is statistical validation of methods, in particular assessment of false positive rates on long interictal data segments [12], driven by the scarcity of long-duration high quality iEEG recordings. The long duration canine iEEG data described in this manuscript represents a valuable asset for assessing the performance of seizure forecasting algorithms, and we are committed to making it available to other investigators. In August, 2014 the American Epilepsy Society and the National Institutes of Health sponsored a seizure forecasting competition through Kaggle.com (http://www.kaggle.com/c/seizure-prediction) using preictal and interictal data clips from these canine data sets. Upon completion of the competition, the full data records and the best performing algorithms will be made publicly available on the International Epilepsy EEG Portal (http://ieeg.org).
