# Correction: Forecasting Seizures Using Bivariate Intracranial EEG Measures and SVM in Naturally Occurring Canine Epilepsy

**DOI:** 10.1371/journal.pone.0156476

**Published:** 2016-05-26

**Authors:** Benjamin H. Brinkmann, Edward E. Patterson, Charles Vite, Vincent M. Vasoli, Daniel Crepeau, Matt Stead, J. Jeffry Howbert, Vladimir Cherkassky, Joost B. Wagenaar, Brian Litt, Gregory A. Worrell

There are errors in the Funding section. The correct funding information is as follows: This work was supported by the National Institute of Neurological Disorders and Stroke at National Institutes of Health (U01-NS073557, R01-NS92882, U24-NS063930), and Mayo Clinic. The authors are grateful for the access provided by NeuroVista to parts of its internal statistical data analysis pipeline. The funding agencies had no part in the design, data collection and analysis, decision to publish, or preparation of the manuscript.
